# A review on over-sampling techniques in classification of multi-class imbalanced datasets: insights for medical problems

**DOI:** 10.3389/fdgth.2024.1430245

**Published:** 2024-07-26

**Authors:** Yuxuan Yang, Hadi Akbarzadeh Khorshidi, Uwe Aickelin

**Affiliations:** ^1^ School of Computing and Information Systems, The University of Melbourne, Parkville, VIC, Australia; ^2^ Cancer Health Services Research, Melbourne School of Population and Global Health, The University of Melbourne, Parkville, VIC, Australia

**Keywords:** over-sampling, re-sampling, multi-class, imbalanced, review, medical

## Abstract

There has been growing attention to multi-class classification problems, particularly those challenges of imbalanced class distributions. To address these challenges, various strategies, including data-level re-sampling treatment and ensemble methods, have been introduced to bolster the performance of predictive models and Artificial Intelligence (AI) algorithms in scenarios where excessive level of imbalance is present. While most research and algorithm development have been focused on binary classification problems, in health informatics there is an increased interest in the field to address the problem of multi-class classification in imbalanced datasets. Multi-class imbalance problems bring forth more complex challenges, as a delicate approach is required to generate synthetic data and simultaneously maintain the relationship between the multiple classes. The aim of this review paper is to examine over-sampling methods tailored for medical and other datasets with multi-class imbalance. Out of 2,076 peer-reviewed papers identified through searches, 197 eligible papers were chosen and thoroughly reviewed for inclusion, narrowing to 37 studies being selected for in-depth analysis. These studies are categorised into four categories: metric, adaptive, structure-based, and hybrid approaches. The most significant finding is the emerging trend toward hybrid resampling methods that combine the strengths of various techniques to effectively address the problem of imbalanced data. This paper provides an extensive analysis of each selected study, discusses their findings, and outlines directions for future research.

## 1 Introduction

### 1.1 Imbalanced data classification

Classification is a key machine learning topic. The primary goal of classification is to accurately assign labels to unseen instances based on learned patterns. In many real-life classification tasks, we often encounter the challenges of imbalanced data.

In the field of medical classification, there are numerous applications for both binary and multi-class classification tasks. In the context of binary classification, one common use is medical diagnostics, where patients are categorised based on the need for surgery. Additional applications of binary classification include detecting cancers, diagnosing diabetes, predicting heart disease, and testing for infections, etc.

For multi-class classification, a common example is the categorisation of surgery risks into multiple levels, based on patient conditions. This method is also used in prognosis of cancer stages, which sits at the scale from Stage I (localised) to Stage IV (advanced) based on factors like tumour size. This method can also be used in identifying diabetes type, predicting stages of heart disease etc.

The following example illustrates how a task can evolve from binary to multi-class classification: In cancer detection, the initial step is to identify whether a patient has cancer. The secondary, more sophisticated task is to determine the cancer's stage. This transition highlights the critical role of multi-class classification in providing detailed and actionable medical assessments.

On the topic of machine learning, the performance optimisation of learning classifiers from imbalanced data has attracted a significant amount of interest in recent years due to the existence of many numerous real-world applications, especially in medical diagnosis (1). As described above, many of the real-world classification tasks face challenges of imbalanced data structure. In the above examples, the likelihood of a “positive” event (“positive prognosis”) is highly improbable relative to the entire data population, resulting in a highly imbalanced dataset distribution. Often, in imbalanced learning scenarios, the minority classes possess high importance and contains additional informational value relative to the majority ones. This discrepancy arises from the skewed distribution of data, where traditional classifiers, assuming a balanced class distribution and equal misclassification costs across classes, fall short. Consequently, these classifiers are prone to being dominated by majority classes, thereby undermining the minority ones. This oversight is greatly amplified in numerous real-world applications, where the attainment of good performance in general proves challenging (2).

In current literature addressing imbalanced dataset, there are 3 main directions, whereby majority of the proposed approaches can be categorised into “algorithmic method”, “ensemble method” and “re-sampling techniques”.

At the algorithmic level, the recognition and determination of minority classes is improved by developing or adapting existing algorithms. These strategies include approaches such as fine-tuning class-specific costs, altering probabilistic estimates at decision tree leaves, re-evaluating decision thresholds, and moving from discrimination-based learning to recognition-based learning with more attention paid on individual classes (3). Cost-sensitive learning is a typical technique under the algorithmic level approach. The crux of its implementation is the underlying assumption in assigning a higher cost to the incorrect labelling of minority class data. It is a versatile approach which allows users to assign different costs to differing scales of minority class within the dataset or at the algorithm level. A typical example is showed in (4), where the study has embedded a cost-sensitive component to the objective function of Neural Network (NN) learners and thereby achieving a statistically significant result of superior performance.

Ensemble methods involve the combination of multiple learners, known as base learners, whereby each is trained to solve the same problem by excelling in different subdomains, thereby complementing each other to achieve superior accuracy (2). With the development of Bagging (5), and Boosting algorithms (6), there have been a great variety of Ensemble algorithms such as improved AdaBoost (7) and SVM Boost (8) proposed to tackle imbalanced data problem. The fundamental strength of ensemble learning is its collective power, significantly surpassing the capabilities of individual base learners. These learners can be derived from any machine learning algorithm, including neural networks, support vector machines, and decision trees, and may be homogeneous or heterogeneous depending on the uniformity of the learning algorithm used. With the evolution of ensemble methods, techniques such as Boosting, Bagging, and Stacking have become central to enhancing the performance of models faced with imbalanced datasets, displaying the versatility and robustness of ensemble approaches in tackling diverse and complex classification challenges (2, 9).

Re-sampling techniques are often employed when dataset is imbalanced with the objective to improve the extent of extremity found in the original dataset. These techniques generally fall into three categories: over-sampling, under-sampling, and hybrid methods. Over-sampling involves inflating the number of instances of the minority class to achieve a more balanced representation of classes. Conversely, under-sampling leads to the removal of majority to attain a balanced dataset. This process often includes the elimination of redundant or noisy data, identified through methods such as K-nearest neighbours (KNN) (10, 11). Hybrid methods combine both over-sampling and under-sampling approaches. By concurrently boosting minority class instances and reducing majority class instances, these methods seek to determine an optimal sampling rate for achieving the most favourable outcome (12).

### 1.2 Re-sampling methods

The scope of this review paper centres on over-sampling techniques. Over-sampling methods can be broadly categorised into two variants: random over-sampling and synthetic over-sampling.

Random over-sampling involves the random selection with replacement of minority class labels within a dataset. Once an instance has been randomly selected, it is duplicated and added into the training dataset. This approach is repeated numerously until the desired ratio of majority and minority class is achieved. This approach is computationally efficient and can be effortlessly implemented as it does not require any inherent knowledge or understanding of the underlying data (13). However, random oversampling is confronted by issues such as overfitting and information loss. This arises because it neglects the underlying structure or distribution of the data, potentially causing the model to learn from noise rather than the true underlying patterns, thus leading to overfitting (7).

To address these drawbacks, synthetic oversampling, such as the SMOTE technique, has been developed. Unlike random oversampling, SMOTE generates new, synthetic minority class instances at the feature level by interpolating between existing minority instances and their nearest neighbours (14). This approach helps to expand the decision boundaries around minority class instances, potentially leading to models that generalise better without merely replicating existing data.

Despite SMOTE's attempt in creating more diverse synthetic data and addressing random oversampling's limitations, issues like noise generation and insufficient data diversity remain. The development of new enhanced algorithms is aimed at generating synthetic data selectively and strategically. These enhancements focus on regions where additional data could most improve model performance. These enhancements result in reduced noise and greater diversity within the synthetic samples, leading to better model accuracy and generalisation in highly imbalanced datasets (15–21).

### 1.3 Multi-class imbalanced data classification

**Figure 1:**
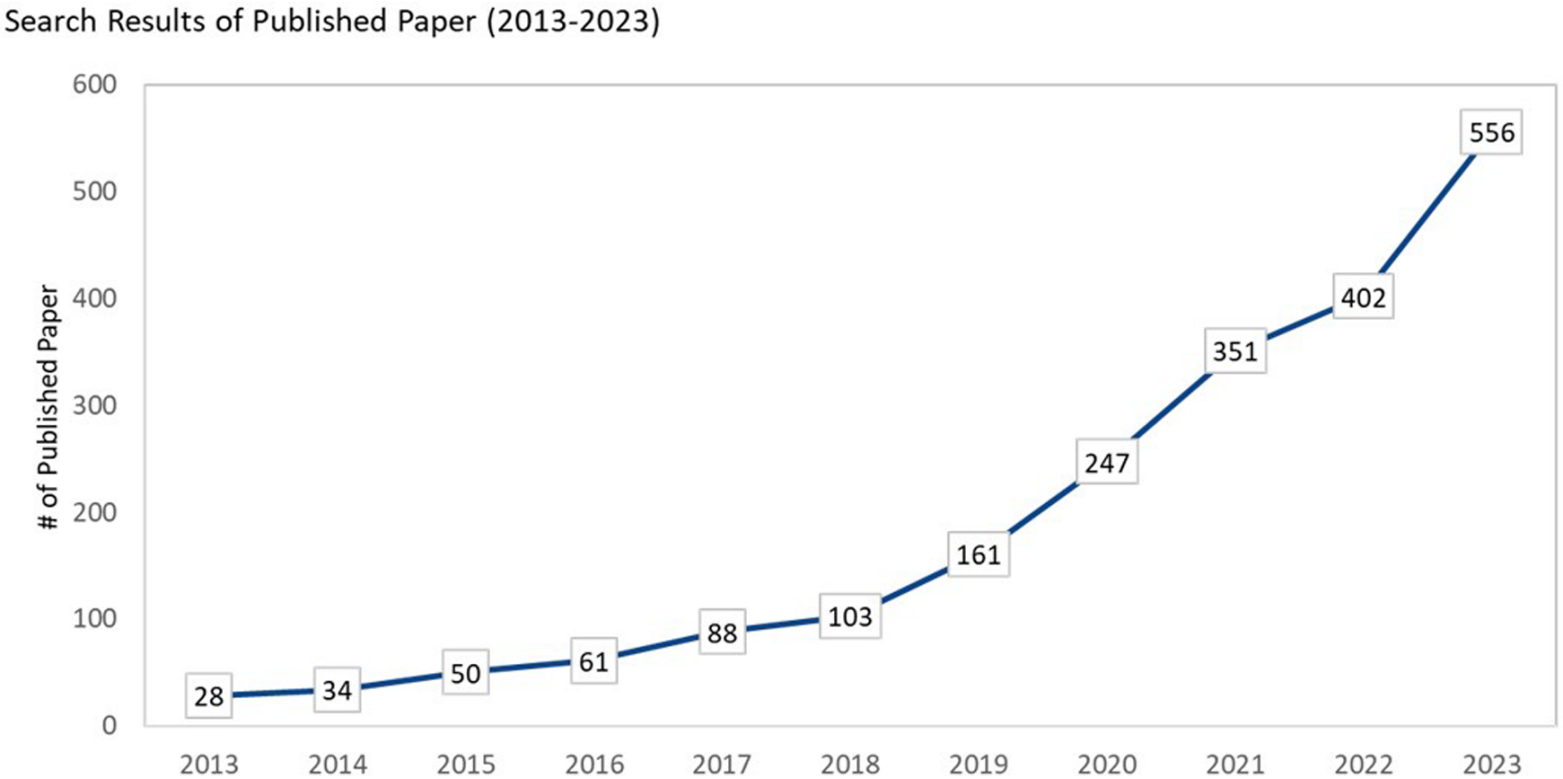
Search results on multi-class classification for imbalance data (search methods are shown in Table 1).

Recently, multi-class classification on imbalanced datasets has garnered increasing attention due to growing demand for multi-class across different settings. Figure 1 illustrate a rising trend in the publication of papers relating to multi-class re-sampling methods.

**Table 1:** Search strategy.

Search	Database	Search query	Year filter
1	ScienceDirect	(“multiclass” OR “multi-class”) AND (“re-sample” OR “resample”) AND (“imbalance” OR “unbalance”)	Year(s): 2013–2023
2	ScienceDirect	(“multiclass” OR “multi-class”) AND (“over-sample” OR “oversample”) AND (“imbalance” OR “unbalance”)	Year(s): 2013–2023
3	Ieeexplore	[(“All Metadata”:“multi-class” OR “All Metadata”:“multiclass”) AND (“All Metadata”:“re-sample” OR “All Metadata”: “resample” OR “All Metadata”:“re-sampling” OR “All Metadata”:“resampling”) AND (“All Metadata”:“imbalanced” OR “All Metadata”:“unbalanced” OR “All Metadata”:“imbalance” OR “All Metadata”:“unbalance”)]	Specify year range: 2013–2023
4	Ieeexplore	[(“All Metadata”:“multi-class” OR “All Metadata”:“multiclass”) AND (“All Metadata”:“over-sample” OR “All Metadata”: “oversample” OR “All Metadata”:“over-sampling” OR “All Metadata”:“oversampling”) AND (“All Metadata”:“imbalanced” OR “All Metadata”:“unbalanced” OR “All Metadata”:“imbalance” OR “All Metadata”:“unbalance”)]	Specify year range: 2013–2023
5	Scopus	TITLE-ABS-KEY [(“multiclass” OR “multi-class”) AND (“re-sampling” OR “re-sample” OR “resample” OR “resampling”) AND (“imbalanced” OR “unbalanced” OR “imbalance” OR “imbalanced”)]	AND (LIMIT-TO (PUBYEAR, 2023) OR [LIMIT-TO (PUBYEAR, 2022) OR LIMIT-TO (PUBYEAR, 2021) OR LIMIT-TO (PUBYEAR, 2020) OR LIMIT-TO (PUBYEAR, 2019) OR LIMIT-TO (PUBYEAR, 2018) OR LIMIT-TO (PUBYEAR, 2017) OR LIMIT-TO (PUBYEAR, 2016) OR LIMIT-TO (PUBYEAR, 2015) OR LIMIT-TO (PUBYEAR, 2014) OR LIMIT-TO (PUBYEAR, 2013)]
6	Scopus	TITLE-ABS-KEY [(“multiclass” OR “multi-class”) AND (“over-sampling” OR “over-sample” OR “oversample” OR “oversampling”) AND (“imbalanced” OR “unbalanced” OR “imbalance” OR “imbalanced”)]	AND (LIMIT-TO (PUBYEAR, 2023) OR [LIMIT-TO (PUBYEAR, 2022) OR LIMIT-TO (PUBYEAR, 2021) OR LIMIT-TO (PUBYEAR, 2020) OR LIMIT-TO (PUBYEAR, 2019) OR LIMIT-TO (PUBYEAR, 2018) OR LIMIT-TO (PUBYEAR, 2017) OR LIMIT-TO (PUBYEAR, 2016) OR LIMIT-TO (PUBYEAR, 2015) OR LIMIT-TO (PUBYEAR, 2014) OR LIMIT-TO (PUBYEAR, 2013)]

Compared to binary-class classification tasks, multi-class imbalance problems involve much more complexity and can be difficult to address relative to typical binary-class imbalance problems (22). Conventional challenges of imbalanced binary-class classification consist of issues such as small sample size and class overlapping, which exist to a greater extent in multi-class problems. This is due to the global presence of such issues among multiple classes. Further challenges arise in multi-class data compared to two-class (binary) imbalanced problems. The most typical challenge is to model the relationships between classes. Multi-class imbalanced data might encounter multiple majority-minority problems. Instances of majority class might be considered as a minority class when compared to a different set of instances, and vice versa (22).

Therefore, compared to binary-class re-sampling, multi-class re-sampling requires better management and mastery in synthetic data generation regions of each class. It is required to have a broadened generation region for minority classes, while controlling the overlap with other classes during the over-sampling process. At the algorithm level, the algorithm should be designed to avoid overgeneration of targeted instances across multiple classes (22).

To address the above challenges, class decomposition is a common strategy employed to address multi-class imbalanced problems. This approach involves translating a multi-class problem into a series of binary subproblems. There are two main decomposition strategies: one-vs.-one (OVO) (23) and one-vs.-all (OVA) (24). Fernandez et al. (25) developed an experimental study and verified the good behaviour of OVO and OVA with re-sampling techniques/ cost-sensitive learning. Various research had been conducted on re-sampling techniques with class decomposition. Combining OVO/OVA with ensemble learning has also shown promising results in recent years. The existing methods mostly use re-sampling techniques in the framework of class decomposition, namely, OVO/OVA. Another way to tackle multi-class imbalanced problem is directly learning from the entire dataset.

### 1.4 Motivation

There have been many studies in the literature which have explored multi-class re-sampling. However, a literature review focusing on these methods has been absent. To the best of our knowledge, while there are plenty of studies reviewing techniques for binary class re-sampling, the unique complexities associated with multi-class re-sampling have not been systematically discussed. For instance, the latest literature review on re-sampling is published in 2023 (26). However, it primarily focuses on binary re-sampling methods, with only two papers discussing multi-class over-sampling. Another survey paper relating to multi-class is introduced in 2023, focusing on classification methods rather than re-sampling techniques (27). This notable gap in the literature highlights the need for a focused review that discusses the specific challenges relating to multi-class re-sampling. This review paper aims to fill this void in the literature by providing a detailed and scholarly review of multi-class re-sampling techniques and therefore enriching academic research with a critical evaluation of this underexplored area.

The rest of this review paper is organised as follows: It begins with the review methodology and search strategy employed to identify relevant papers. This is followed by a comprehensive review of the literature, organised into four categories: metric, adaptive, structure-based, and hybrid. Subsequently, the discussion section delves into the analysis of each category, accompanied by citation and trend analysis. The paper concludes with recommendations for future research directions.

## 2 Methods

### 2.1 Eligibility criteria

Eligible primary studies were required to satisfy the following criteria: (1) the proposed algorithm must be specifically designed for imbalanced multi-class classification. This implies algorithms primarily designed for binary class and subsequently applied to multi-class use cases are excluded. (2) similarly, application of existing imbalanced multi-class algorithms is excluded. (3) the proposed algorithms must not be related to multiple ordinal labels, as it requires a completely different treatment.

### 2.2 Information sources

Candidate studies were identified by searching the following electronic database. Scopus, Science Direct, and IEEE Explore. The search strategies were designed and originally conducted on Jan 2023, with the final search conducted in Jun 2024.

### 2.3 Search strategy

The search strategy (Table 1) for each database was constructed from the corpus of keyword terms that are related to the following: multi-class; over-sampling; imbalance. To ensure recency and relevance, we've set the year filter to the most recent decade (2013–2023).

### 2.4 Data charting and extraction

For each relevant article, we extracted information about the study characteristics (publication year, type), and abstract. For articles after initial abstract screening, we extracted information about the methodology, experimentation, and main contributions.

## 3 Results

### 3.1 Summary of sources of evidence

**Figure 2:**
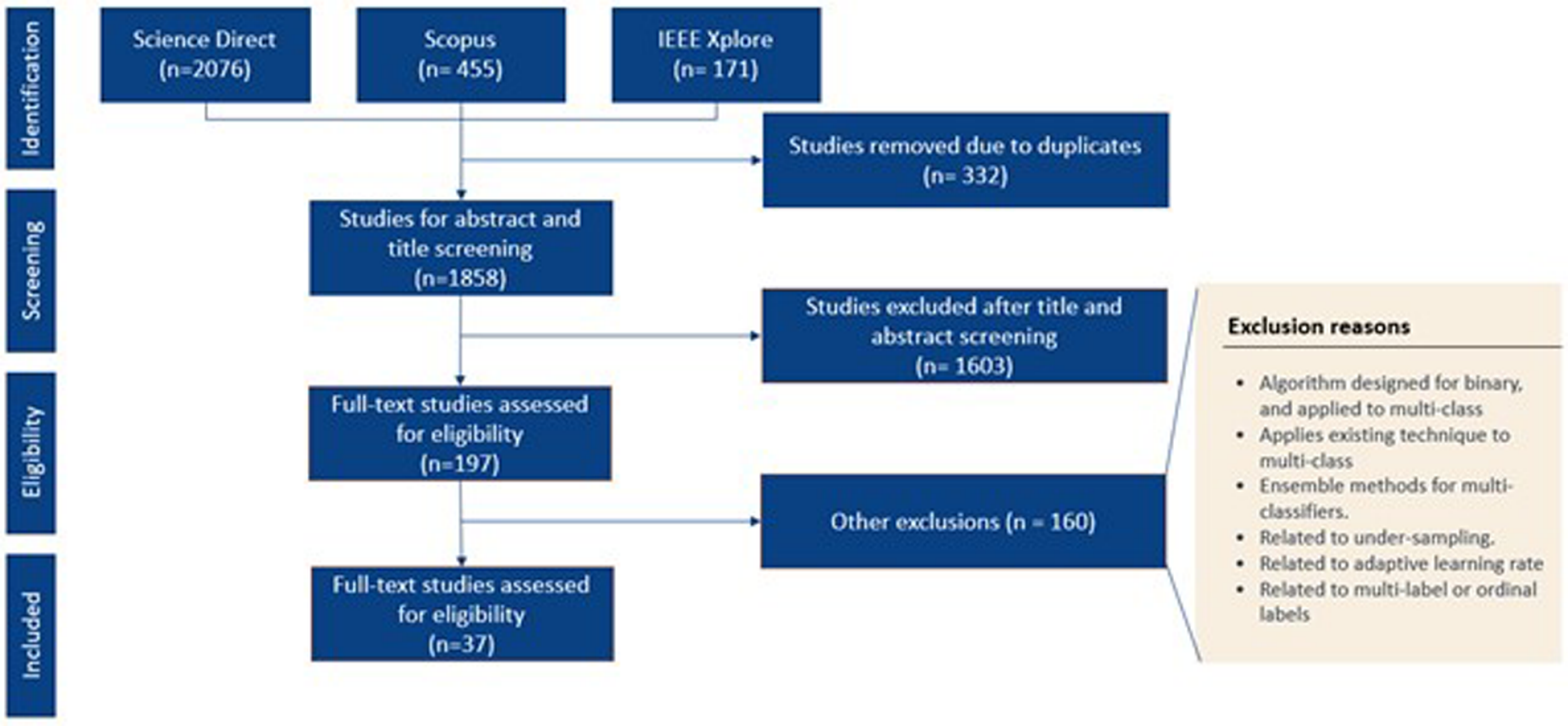
Searching workflow.

As shown in Figure 2, our searching workflow yielded 2,076 peer-reviewed paper. Among them, 197 full-text papers were assessed for final eligibility, which resulted in 37 eligible studies for synthesis analysis.

All papers were published after year 2016, with 75% published between year 2018 and 2023. This reflected increasing awareness to address multi-class imbalanced re-sampling. In terms of regional distribution, most of the eligible studies was primarily conducted in East Asia (40%), followed by cross-regional collaboration (24%) and Poland (13%).

**Figure 3:**
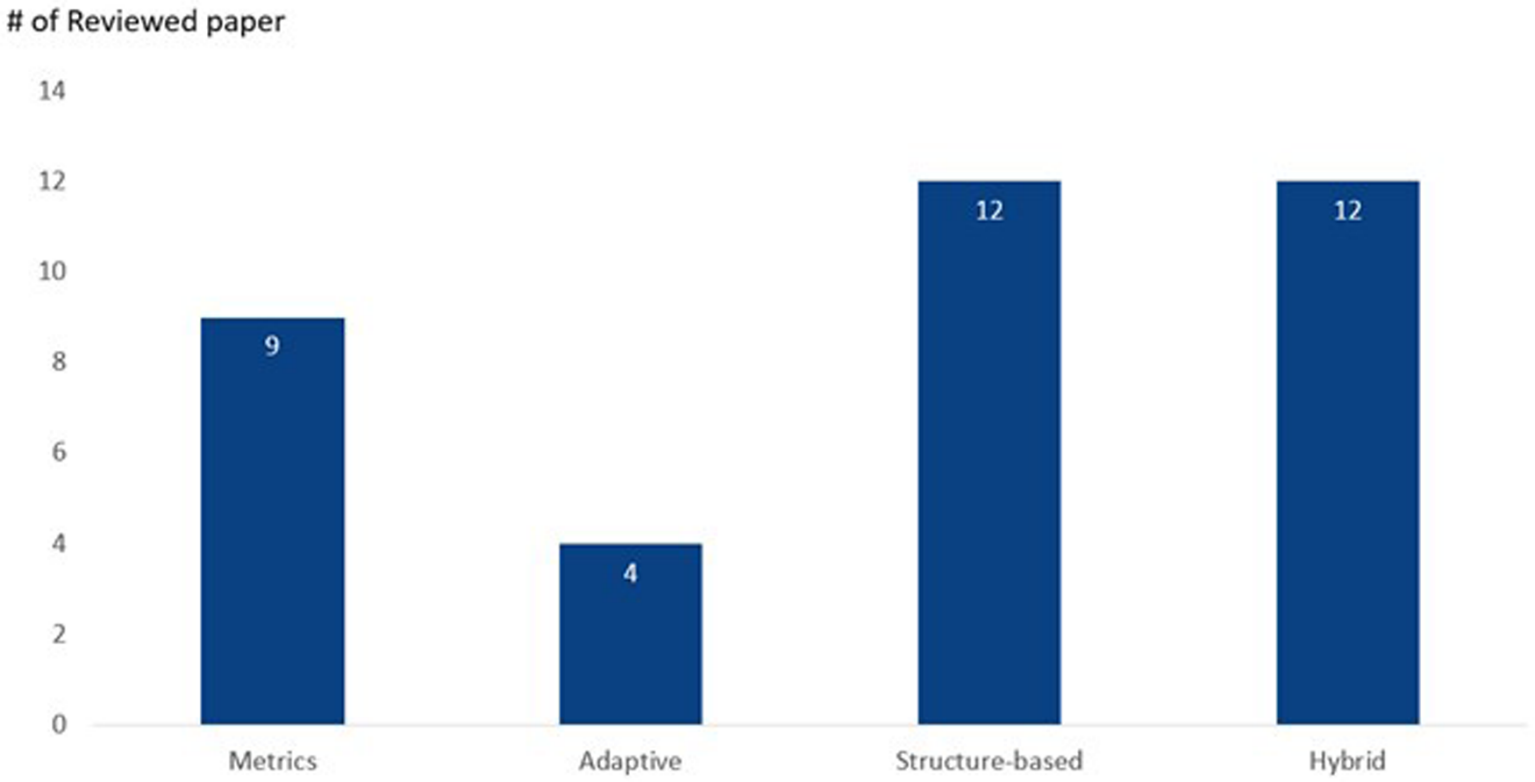
Topic categorisation.

As shown in Figure 3, the 37 relevant research paper is then categorised in the following categories: Metrics, Adaptive, Structure-based, and Hybrid.

The description for each category is provided in Table 2. It is important to note that the categories outlined are not mutually exclusive, a single paper may fit into more than one category. In cases where this occurs, we assign the paper to the most relevant category.

**Table 2:** Description of topic categorisation.

Category	Description
Metric	Uses specific distance or similarity metrics to guide over-sampling.
Adaptive	Dynamically adjusts over-sampling based on specific conditions or attributes in the dataset.
Structure-based	Focuses on the inherent structure or distribution of the data, analysing patterns, boundaries, or relationships.
Hybrid	Combines elements from multiple over-sampling techniques, classifiers, binarisation, and re-sampling strategies,

The summary of each paper is provided in Table 3, including the area of application, source of data, number of datasets, algorithm methods, splitting strategy, ML techniques, year of publication, region of the author, and number of citations as of 19th June 2024. This table is adopted from Mei et al. (64).

**Table 3:** Details of each paper.

Category	Area of application	Source of data	# of dataset	Algorithm methods, splitting strategy, cross validation	ML techniques	Year	Region	Reference	# of Citation as of 19th June 2024
Metric	Real-life imbalanced class scenario	UCI KEEL	5	Comparison of proposed technique (Hellinger distance-based oversampling) against MDO, SMOTE, ADASYN	KNN, C4.5	2017	India	(28)	13
Metric	Real-life imbalanced class scenario	KEEL	20	Comparison of proposed DRCW-ASEG with state-of-the-art methods (OVO-Easy, AdaBoost.NC, Static-SMOTE)	CART	2018	China, Spain, Saudi Arabia	(29)	37
Metric	Real-life imbalanced class scenario	KEEL	20	Comparison of proposed method (MC-RBO) against SMOTE-all, S-SMOTE, MDO, SMOM, NB-SVM, AdaB.NC, SMOTEBag, OVO-RUS, OVO-SMOTE, OVO-BSMOTE, OVO-ADASYN	C5.0 Decision trees Multilayer Perceptron Lazy learners (k-NN), Naïve Bayes	2019	Poland	(30)	96
Metric	Medical classification/Clinical Diagnostic	KEEL	20	Comparison of proposed algorithm (MC-CCR) against SMOTE-all, S-SMOTE, MDO, SMOM, SMOTE-IPF	C5.0, Multilayer Perceptron (MLP), k-NN	2020	Poland & USA	(31)	106
Metric	Credit Scoring	Various (UCI, Kaggle etc.)	7	Comparison of proposed method (cWGAN) against SMOTE, B-SMOTE, ROS, SMOTENC (SMOTE-Nominal Continuous), ADASYN	RandomForest, Logistic Regression, Gradient Boosting, k-nearest-neighbours (KNN), decision tree	2021	Germany	(32)	192
Metric	Network traffic	UCI NSL-KDD resampled from the earlier KDD cup99 dataset (Tavallaee et al., 2009) Australian Cyber Security Centre	3	Comparison of proposed algorithm against SMOTE, MDO, SOUP	Logistic Regression, Bayes Classifier	2022	China	(33)	0
Metric	Real-life imbalanced class scenario	UCI KEEL	20	Comparison of proposed method MDO against ROS, SMOTE, B-SMOTE, ADASYN	C4.5 Decision Tree, KNN, Ripper	2016	Iran	(34)	378
Metric	Real-life imbalanced class scenario	UCI KEEL	15	Comparison of proposed method AMDO against MDO, MDO+, S-SMOTE, AdaBoost.NC, OVO-SMOTE	C4.5 Decision Tree	2018	China	(35)	74
Metric	Real-life imbalanced class scenario	UCI KEEL	9	Comparison of proposed method (EMDO) against S-SMOTE, GCS, AdaBoost.NC, OVO-SMOTE, MDO, MDO+, AMDO	C4.5 Decision Tree	2021	Taiwan	(36)	9
Adaptive	Real-life imbalanced class scenario	UCI KEEL	15	Comparison of proposed method against SMOTE, KMOTE, SWIM, ASUWO, BorSMOTE, ADASYN, MWMOTE etc.	k-NN, Decision Trees	2021	China	(37)	65
Adaptive	Real-life imbalanced class scenario	17 (KEEL) 9 medical datasets (Broad Institute)	26	Comparison of proposed AFNFS against 6 other algorithms (SKNN, EUSAB, RUSAB, S2SML, KS2SML, US2SML) on 17 KEEL datasets	KNN, C4.5, SVM	2022	China	(38)	21
Adaptive	Real-life imbalanced class scenario	UCI	21	Comparison of proposed method (OREM-M) against SMOTE, SMOM, MDO, MC-RBO	C4.5, NN, SVM	2022	China	(39)	19
Adaptive	Network traffic/intrusion detection	NSL-KDD resampled from the earlier KDD cup99 dataset	1	Comparison of proposed method (SMMO-CoFS) against SMOTE, CoFS	RandomForest, J48, BayesNet, AdaBoostM1	2023	China, Ethiopia	(40)	3
Structure-based	Real-life imbalanced class scenario	8 from ordinal regression literature 19 from UCI	27	Comparison of proposed method (SMOM) against ROS, SMOTE, ADASYN, SL-SMOTE, MWMOTE, MDO	C4.5, AdaBoost (C4.5), MLP, NB	2017	China & US	(22)	234
Structure-based	NoSQL BigData scenario	8 from UCI 1 pharmacology-based 1 from ProgrammableWeb	10	Comparison of proposed method (UCPMOT) in conjunction with MEMMOT, MMMmOT, CMEOT, NF_N (Nearest Farthest Neighbour) against SMOTE, Borderline-SMOTE, ADASYN, SPIDER2, SMOTEBOOST, MWMOTE	RandomForest, Naïve Bayes, AdaBoostM1, MultiLayer Perceptron	2017	India	(41)	17
Structure-based	Medical classification/Clinical Diagnostic	Empirical medical data from France	1	Comparison of proposed method (SOMCID) against Multi-Class Random Oversampling (MCROS), Static-SMOTE, AdaBoost.NC	C4.5, Decision Trees	2018	Spain, USA, Poland	(42)	2
Structure-based	Medical classification/Clinical Diagnostic	Medical, source unspecified	1	Comparison of proposed technique (Mudiot) at different parameters *k*	Not specified	2019	India	(43)	2
Structure-based	Real-life imbalanced class scenario	KEEL UCI OpenML	11	Comparison of proposed algorithm OSC (Oversampling with Spectral Clustering) against OSM, IAOS, OUB, Multi-IM, DRCW-ASEG, OEE, OSB	CART Decision Tree	2020	China & USA	(44)	45
Structure-based	Medical classification/Clinical Diagnostic	UCI datasets (Medical + non-medical) and private missed abortion datasets	13	Comparison of proposed KNSMOTE to 6 traditional oversampling algorithms (SM, BSM, ADA, ANS, MDO, GSM) and 4 other cluster-based oversampling Comparison of proposed KNSMOTE with 3 different ensemble classifiers	C4.5, Ensemble methods (Adaboost, Bagging and RF)	2021	China	(45)	125
Structure-based	Real-life imbalanced class scenario	UCI	5	Comparison of proposed method (STCPS) against SMOTE, SVMOM, SMO + TLK, SVM + ENN	Not explicitly stated, but SVM + ENN uses RandomForest	2021	China	(46)	15
Structure-based	Medical classification/Clinical Diagnostic	Empirical data sourced from human-machine scenario	3	Comparison of proposed algorithm PB-SMOTE against SMOTE	Neural networks (MLP)	2021	Spain	(47)	11
Structure-based	Real-life imbalanced class scenario	UCI	25	Comparison of Proposed method (PAIO) against ROS, SMOTE, SL-SM, MWMO, SMOM, INOS	C4.5, SVM	2022	China	(48)	71
Structure-based	Real-life imbalanced class scenario	UCI	4	Comparison of proposed method (cross-sampling procedure) against pre cross-sampling	Decision Trees, KNN, Logistic Regression, Naïve Bayes, RandomForest, SVM	2023	Indonesia	(49)	0
Structure-based	Real-life imbalanced class scenario	UCI Multi-imbalanced package (python)	15	Comparison of proposed method (GMMSampling) against RUS, All-kNN, ROS, K-means SMOTE, density estimation (KDE), MC-CCR, SOUP, MDO etc.	CART Decision Trees, Naïve Bayes, k-nearest neighbours (KNN), RandomForest, Gradient Boosting Trees	2023	Poland	(50)	0
Structure-based	Real-life imbalanced class scenario	UCI KEEL	10	Comparison of proposed method (SMOTE-IF) against SMOM, Noise Reduction A Priori Synthetic Over-sampling (NRAS), CURE_SMOTE, K-means SMOTE, BoostOBU	KNN (k-Nearest Neighbour)	2023	China	(51)	0
Hybrid	Real-life imbalanced class scenario	UCI	21	Comparison of proposed framework (oversampling of selective classes) on datasets with no preprocessing and full preprocessing of all classes	C4.5, SVM, KNN	2016	Poland	(52)	250
Hybrid	Real-life imbalanced class scenario	UCI KEEL	7	Comparison of proposed algorithms (MEMMOT, MMMmOT, CMEOT) to SMOTE, SafeLevel SMOTE, ROS	RandomForest, Naïve Bayes, AdaBoost	2017	India	(53)	18
Hybrid	Real-life imbalanced class scenario	15 from UCI 4 from synthetic datasets derived in previous research	19	Comparison of proposed algorithm (SOUP) against Static-SMOTE, Global CS, OVO-ROS, OVA-ROS, OVO RUS, OVA RUS, OVO NCR, OVA NCR, OVO SO, MRBB (Multi-class Balanced Bagging)	PART, J48, kNN	2019	Poland	(54)	52
Hybrid	Real-life imbalanced class scenario	KEEL	20	Comparison of One vs One, One vs All, Error Correcting Output Codes	Naïve Bayes, k-nearest Neighbours, CART, SVM, Logistic Regression	2020	Poland	(55)	5
Hybrid	Fault diagnosis recognition	Case Western Reserve University (CWRU) Intelligent Maintenance Systems (IMS)	14	Comparison of proposed algorithm (SCOTE) against ROS, BSMOTE, SMOTE, A-SUWO, ADASYN, Cluster SMOTE, MWMOTE Comparison of proposed combination of SCOTE with Least-Squared SVM (SCOTE LS-SVM framework) against other ML algorithms	AdaBoost.M1, SAMME, ADAC2.M1, ADABOOST.NC, FuzzyImbECOC, HDDTOVA, MCHDDT, ImECOC	2021	China	(56)	111
Hybrid	Real-life imbalanced class scenario	KEEL	27	Comparison of proposed method DPSE against OVA scheme (SMOTE, k-means SMOTE, Bagging-RB, OVA (without sampling) Comparison of proposed method DPSE against OVO scheme (DES-MI, OVO-SMB, OVO-EASY, OVO-DPSE)	CART, Random Forest, SVM	2021	China	(57)	24
Hybrid	Real-life imbalanced class scenario	OpenML Datasets Repository	5	Comparison of proposed algorithm (ADASYN with stacking) against original dataset, OSS SMOTE	SVM, Random Forest, Stacking	2022	Indonesia	(58)	6
Hybrid	Real-life imbalanced class scenario	KEEL	20	Comparison of proposed method (noise-robust oversampling) against SMOTE-all, S-SMOTE (Static-SMOTE), MDO, SMOM, MC-CCR, MC-RBO etc.	C5.0, MLP, KNN, Naïve Bayes	2023	China	(59)	35
Hybrid	Real-life imbalanced class scenario	KEEL	11	Comparison of proposed method, Ensemble-based re-sampling method (E-EVRS) against RUS-Bagging, RUSBOOST, SMOTE-ALL, S-SMOTE, MDO	Decision Trees, SVM	2023	France, Tunisia	(60)	5
Hybrid	Real-life imbalanced class scenario	UCI KEEL	11	Comparison of proposed method (OOSI) against MWMOTE, Kmeans-SMOTE, G-SMOTE, DBSMOTE, ADASYN etc.	Logistic Regression, SV, Adaptive Boosting, Gradient Boosted Trees, Neural Networks (Backpropagation)	2023	China	(61)	0
Hybrid	Fault diagnosis recognition	Turbine data from Ireland	1	Comparison of proposed method AdaptiveSMOTE with edited nearest neighbours (ASMOTE-ENN) against original data, SMOTE, SMOTE with Tomek links, B-SMOTE, Kmeans-SMOTE-ENN, Clustering SMOTE	Decision Trees, KNN, AdaBoost, RandomForest	2023	South Korea	(62)	7
Hybrid	Real-life imbalanced class scenario	NSL-KDD resampled from the earlier KDD cup99 dataset UNSW-NB15 KDD Cup 1999 CICIDS2017	4	Comparison of proposed 3 stage data generation algorithm (SMOTE-GAN-VAE) against SMOTE-GAN (Generative adversarial network), SMOTE-VAE (variational autoencoder), GAN-VAE	SVM	2023	China, UK, UAE, Jordan, Saudi Arabia, India, Lebanon	(63)	3

### 3.2 Metric

Metric techniques are centred around specific distance or similarity metrics. They leverage measures like Hellinger distance, Evolutionary Mahalanobis Distance, or other metrics to inform and guide the over-sampling process, emphasising distances or differences between data points. There are 9 papers classified under this category.

In 2017, Kumari and Thakar propose a 2-step distance based over-sampling method (28). The novelty comes with the introduction of Hellinger's distance in the second step. Hellinger's distance is based on Hellinger's integral, which is introduced by Ernst Hellinger in 1909. The first step is conducted using k-nearest neighbours using Euclidean distance that identifies samples to be used for re-sampling. The 2nd step is the over-sampling algorithm (referred to as “Dataset Balancing Module”), which determined the population of synthetic data in the appropriate minority data region using Hellinger's distance. It achieves this by directing the generation of synthetic samples towards areas with a higher concentration of neighbours within the same class, thereby avoiding the blending of minority and majority class regions. The authors noted that the algorithm can be extended to address within-class imbalanced data, and to investigate expanding it to multimedia data.

DRCW-ASEG (29) is proposed by Zhang et al. in 2018 as an algorithm to enable the creation of synthetic minority class instances using a dynamic weighting procedure. The DRCW-ASEG algorithm can minimise bias towards majority classes. The proposed algorithm works by a 2-pronged process. In the initial or learning phase, the multi-class is sub-divided into easier-to-address binary classification problems using OVO strategy. In the second phase, non-competent classifier problem is addressed using an aggregation method. DRCW-ASEG works by an adaptive process which helps to generate synthetic instances in the local region, which differs slightly to SMOTE, to help balance the data distribution. Heterogeneous Value Difference Metric (HVDM) is used as the preferred measure to calculate distance between 2 instances, and it allows for numerical and nominal attributes.

A Combined Cleaning & Re-sampling algorithm is proposed by Koziarski et al. in 2020 (31). It enables targeted minority over-sampling which exploits local class characteristics by using knowledge of the inter-class relationship in multi-class setting. There are two algorithms, namely Binary Combined Cleaning and Re-sampling (CCR) and Multi-Class Combined Cleaning and Re-sampling (MC-CCR). They proposed 3 stages of the algorithms, including forming sphere around minority instances, tidying up majority instances within the spheres, and conducting adaptive over-sampling accordingly on the spherical radius. The spherical region is conducted using an energy-based approach around every minority observation. The expansion of each sphere is dependent on the energy, which is an input parameter of the proposed algorithm. The data synthesis step is then conducted within the spherical region, with the size of the calculated sphere used to determine the weighting of minority instances. The relative distance of the instances plays an important role in determining the weighting in contrast to ADASYN.

Krawczyk et al. proposed a radial-based over-sampling method in 2020 (30). The paper introduces a new multi-class over-sampling algorithm called MultiClass Radial-based over-sampling (MC-RBO). The paper also introduces a metric or term called “potential”, which is referred to as the cumulative closeness of a particular point to a collection of observations. The potential of a point increases if it is located nearby many observations in the same collection. In this paper, the potential function is defined using Gaussian Radial Basis function (RBF). There are 2 unique areas about MC-RBO. Firstly, it utilises information from all classes to generate synthetic instances, in contrast to the many existing multi-class over-sampling algorithms which focused heavily on minority class. Secondly, the method is suitable for unusual data distributions, as it relies on the generation of synthetic instances in regions where mutual class potential is close to zero. Mutual class potential is defined as the disparity in potential between 2 different classes and is utilised to assist with the identification of regions of overlap between classes or regions of high uncertainty. In its binary form, B-RBO generates synthetic samples in binary classification by targeting regions of high uncertainty, where mutual class potential is near 0. MC-RBO builds on this for multiclass problems, iteratively oversampling each minority class against all other classes, sorted by number of observations per class. This approach ensures balanced class representation and reduces computational load.

Abdi & Hashemi introduce Distance-based Over-sampling (MDO) (34), an algorithm which integrates Mahalanobis Distance for over-sampling in multi-class imbalanced datasets. MDO generates synthetic data points with equivalent Mahalanobis distance from the class mean as other minority class examples, thereby preserving the covariance structure of minority instances. By generating synthetic samples within each minority region, MDO improves the modelling of minority class instances for learning algorithms and minimise the risk of overlapping between different class regions.

In 2016, Yang et al. introduce Adaptive Mahalanobis Distance-based Over-sampling (AMDO) (35). AMDO is an extension of MDO and it accommodate mixed-type datasets by integrating Generalized Singular Value Decomposition (GSVD) (65) and a distance metric, the Heterogeneous Value Difference Metric (HVDM) (66). This enables effective handling of both numeric and nominal attributes. It also incorporates a partially balanced resampling concept, which selectively over-samples minority classes based on their size and the desired final distribution to mitigate over-generation risks, thus ensuring a more effective synthetic sample generation which does not compromise the majority class's accuracy.

In 2021, Yao & Lin (36) introduce an over-sampling approach called Evolutionary Mahalanobis Distance Over-sampling (EMDO), which allows for the application of multiple ellipsoids in parallel. This addresses the challenges in MDO (34) and Adaptive Mahalanobis Distance-based Over-sampling (AMDO) (35), where their decision regions may overlap for ring-belt-shaped regions due to the presence of only 1 ellipsoid. This limitation is addressed by integrating the usage of Gustafon-Kessel Algorithm (GKA) with multi-objective particle swarm optimisation (MOPSO). The proposed EMO allows for better approximation of the complex data regions, enabling it to be better applied on multi-class imbalanced dataset.

Engelmann and Lessmann propose Conditional Wasserstein GAN-based oversampling of tabular data for imbalanced learning to implement Conditional Wasserstein Generative Adversarial Network (CWGAN) with a Gradient Penalty (GP) to tackle imbalanced tabular datasets (32). CWGAN algorithm ensures appropriate and targeted generation of synthetic minority instances to improve minority class representation. The gradient penalty serves as a regularisation tool, ensuring that a discriminator trained to its optimal state exhibits a smooth, linear gradient. This not only guides the generator towards the data distribution but also limits the capacity of the discriminator. The incorporation of Gradient Penalty is accounted for the common problems such as convergence and mode collapse (i.e., same types of data which are repeatedly produced) and provided improved stability.

Zhao et al. propose a method to tackle the issue of data imbalance, specifically in the setting of network traffic in convergence network in 2022 (33). It involves sample selection and subsequent data synthesis process. The sample selection process is conducted using the measure of density information of each class vs. the individual information of each sample instance within the specific class. The sample weight for each instance is then determined based on the information content of the sample. This process is repeated until the information gap is greater than 0. The data synthesis process is conducted using eigenvalue decomposition through Principal Component Analysis (PCA). The authors specifically pointed out that Euclidean distance was not used for synthesis, stating that linear interpolation will cause covariance structure of features to change in multi-class datasets.

### 3.3 Adaptive

Adaptive techniques dynamically adjust the over-sampling based on certain conditions or attributes within the dataset. They might consider variance, distance, or other specific characteristics to determine their over-sampling strategy, making them flexible in nature. This concept, which has been gaining attention since 2020, is notable for its flexibility. There is a total of 3 papers which are classified under this category.

The paper by Wang et al. introduces the Local distribution-based Adaptive Minority Over-sampling (LAMO) algorithm, an adaptive approach designed to address challenges in imbalanced datasets through effective oversampling (37). LAMO dynamically adapts its oversampling strategy by identifying borderline minority instances using a kd-tree and Euclidean distance. Subsequently, it generates synthetic minority class instances using a Gaussian Mixture Model (GMM) to model the probability distribution of the minority class, thereby tailoring the oversampling process to the local data distribution. The adaptability allows LAMO to effectively address the challenges of imbalanced datasets by focusing on regions where oversampling is most needed, thereby improving its performance in handling class imbalance compared to non-adaptive techniques.

An adaptive synthetic over-sampling method (AFNFS) is proposed by Sun et al. in response to addressing concerns on the classification efficiency for imbalanced data (38). The proposed algorithm works in 3 key steps. Firstly, the variance distance between minority class instances was proposed as a measure of “closeness” and this set is further specified to be an extrapolation technology based on Gaussian distribution. This is then used to construct the over-sampling model to obtain a balanced environment of synthetic and original instances. The second step is to focus on the development of fuzzy neighbourhood radius, which is specified based on the margins of similar and dissimilar instances. The neighbourhood radius is developed adaptively. The corresponding fuzzy neighbourhood granule, degree of membership, and upper and lower boundaries are then used to design our FNRS model. Lastly, by combining the outcome from the previous 2 steps, joint entropy with roughness can be used to construct fuzzy neighbourhood decision systems to evaluate uncertainty. This adaptive method addresses concerns regarding imbalanced data classification by tailoring oversampling techniques to local data distributions, potentially improving classification accuracy and model performance.

Zhu et al. propose novel re-sampling techniques using an adaptive concept, introducing the OREM algorithm, which is extended to handle multi-class imbalanced datasets (OREM-M) (39). OREM identifies candidate minority regions (CMRs) based on proximity and majority distribution, then generates synthetic minority instances within clean sub-regions to ensure reliability. OREM-M addresses multiclass imbalance, ensuring no overlap between minority classes during synthetic instance generation. Additionally, OREMBoost integrates OREM with boosting to enhance classifier learning by balancing datasets through oversampling at each iteration. The entire process repeats based on the number of iterations T decided. Overall, the final classifier is constructed based on each of the previous classifiers built, and the weightage assigned is based on their performance. This process of OREM provides an approach to solving the multi-class imbalanced problem.

In 2023, Damtew et al. propose a Synthetic Multi-minority Oversampling technique (SMMO) (40). It is an adaptive re-sampling technique that incrementally generates new minority instances. The acceptance of the synthetics instances is determined by the distance to nearest minority and majority data points. Once accepted, it is incorporated into the subsequent data generation cycle. This technique is specifically designed to reduce data overlaps among multiple classes.

### 3.4 Structure-based

These methods revolve around the inherent structure or distribution of the data. Techniques often involve clustering or grouping data points, especially minority samples, to decide on the generation of synthetic samples. The focus is on identifying “safe” regions or segments within the data. While it might employ clustering as one of the techniques, it also considers other attributes like data density, boundary behaviour, or data hierarchies. It is broader in its understanding of how the data is organised. The secondary contribution of the paper also sought to extend existing over-sampling techniques for binary classification to ordinal regression.

A Synthetic Minority over-sampling technique, SMOM, is published in 2017 by Zhu et al. (22). This approach, falling under the structure-based category, utilises a k-nearest neighbours (k-NN) method to generate synthetic instances with allocated selection weights based on the direction of nearest neighbours. Unlike random allocation, this weighted approach aims to reduce over-generalisation by placing more emphasis on safer, suitable synthetic instances. SMOM employs Neighbourhood-Based Clustering for Outstanding Instances (NBDOS) to distinguish between “outstanding” and “trapped” minority instances, assigning selection weights accordingly. It offers superior performance due to the effectiveness in broadening the minority class region while mitigating overlap between different minority classes, offering a promising direction for handling imbalanced datasets with nominal and ordinal attributes in the future.

Saez et al. propose Selective Oversampling for Multi-class Imbalanced Datasets (SOMCID) framework for multi-class imbalanced challenges in medical application of vertebral column pathologies classification (42). It first classifies each instance into one of the 4 categories (i.e., safe, borderline, outlier, or rare) based on the local region of each instance and their proximity to other classes. HVDM (Heterogenous Value Difference Metric) is used to measure the distance between instances. The next step involves the actual minority classes to apply oversampling and types of instances which should be used for oversampling within the respective minority class. This will result in multiple oversampling “configurations”, which are different combinations of classes and instances to be oversampled to reach the optical outcome to balance dataset efficiently. Oversampling technique (e.g., SMOTE) can then be applied until class distribution is balanced. This method aims to relieve the issue of skewed class distribution by focusing only on specific instances.

In 2019, Li et al. proposed a method to solve the imbalanced problem of multiclass classification (44). The proposed technique incorporated OVO decomposition to first decompose multi-class datasets into binary class datasets, and subsequently applying spectral clustering to partition minority classes into subspaces. The over-sampling or generation of synthetic minority instances is then conducted accordingly to the characteristics of each sub-space, utilising information from data distribution, and thus avoiding over-sampling outliers.

Zhu et al. in 2020 propose an interpolation-based over-sampling techniques (48). The authors introduce Position characteristic-aware interpolation over-sampling method (PAIO) to synthetically generated minority instances, called Generalisation Interpolation Creation (GIC), a synthetic generation method. PAIO was introduced to overcome the challenges of interpolation-based over-sampling, such as the over-constraint on inland minority instances, low-efficiency expansion of the minority samples at the borderline region and the over-generalisation of trapped minority instances.

A technique that aims to update class purity for over-sampling (UCPMOT) is proposed in 2017 (41). This technique is proposed to generate synthetic samples based on exclusive safe levels based on KNN.

An algorithm that combines SMOTE and k-means, named KNSMOTE for imbalanced medical data is proposed Xu et al. in 2021 (45). KNSMOTE works firstly by the application of k-means clustering on the original dataset. A filtering strategy is then conducted based on class difference calculations of instances on the clustered instances to identify “safe” samples, which allows a successful way of deleting boundary, overlapping and noisy samples while simultaneously retaining the integrity of important instances. Finally, the “safe” instances which were previously identified are then linearly interpolated with SMOTE to generate 2 new classes of synthetised instances. To address the possibility that the classes of the instances may have changed after the cluster-based filtering strategy, sampling ratios are configured based on imbalanced ratio factoring, (1) the scenario where conversion of majority to minority class instances did not occur, and (2) the scenario where conversion of majority to minority class instances do occur.

Deng et al. introduce an innovative technique utilising hyperplanes to address multi-class imbalanced datasets in 2021 (46). This approach involves sorting data within each class based on their distance relative to the hyperplane and performing iterative intra and inter-class sampling at the decision boundary of neighbouring classes. By incorporating data density and sorting information, the algorithm generates synthetic instances while preserving the characteristics of neighbouring instances. By incorporating data density and sorting information, the algorithm generates synthetic instances while preserving the characteristics of neighbouring instances.

Leteifa and Torres introduce Perceptual Borderline Oversampling (PBO) and Perceptual Borderline SMOTE (PB-SMOTE) (47). PB-SMOTE operates by projecting samples into the relevant space, and then applies a threshold calculation to determine borderline vs. non-borderline minority instances. The technique also allows for different pre-determination of nearest neighbours to be used for interpolation of synthetic instances. It is able to populate more diverse and accurate minority instances near the boundary of majority regions, thereby helping classifier model to improve learning on the complex boundaries.

Multiclass Data Imbalance Over-sampling Techniques (MuDIOT) is introduced in the literature (43). The proposed technique identifies obscure factors which have negative performance impact on classification, and then seeks to minimise issues caused by imbalanced data by re-balancing the data. MuDIOT is based on SMOTE but it takes k-nearest neighbours into consideration for each instance, calculating distance between centroid and instance, and then subsequently applying various adjustments such as magnitude of the difference between instances. These subtle differences are then added onto the original instance to create synthetic instances. The author concluded that experimental results showed an increase in performance and produce better classification accuracy.

Cross-Sampling (CS) method is proposed in 2023 (49). The technique is focused on data pre-processing to prevent over-lapping classes. It starts with using clustering method to identify the structure of the classes and form clusters. Temporary labels are created based on pattern matching. Subsequent evaluations of temporary labels are conducted against the original label, retaining only those that are true positives, so that all the “confusing” instances are removed. Following this cleaning process, SMOTE is incorporated to generate new instances.

GMMSampling was introduced in the same year (50). It fits a Gaussian Mixture Model to each minority class to understand the distribution. Synthetic samples are generated according to the estimated class distribution. The synthetic samples are completely new instances, rather than mere modification of existing data points. During the generation process, GMMSampling prioritises regions with the most unsafe minority classes, guided by calculated safe levels. This approach enhances the informativeness and diversity within the dataset.

Again, in 2023, Li et al. introduced SMOTE-IF (51), a resampling method that combines SMOTE with a variant of Isolation Forests to tackle challenges in multi-class imbalanced data classification. SMOTE-IF first generates synthetic data points using SMOTE, followed by incorporating Isolation Forest technique to identify and remove erroneous samples. Isolation Forest uses binary trees to detect anomalies, which helps to prevent the excessive generation of minority class samples towards the majority class region, thereby improving classification accuracy.

### 3.5 Hybrid

Hybrid combine elements or concepts from multiple over-sampling techniques or approaches. They integrate diverse strategies, often blending the best of multiple methods to address the imbalanced data problem.

A hybrid approach, combining the SCOTE algorithm with LS-SVM is proposed in 2020 by Wei et al. (56). This hybrid approach incorporates SCOTE to remove noisy instances, computes the importance of minority instances using (Least Squared Support Vector Machine LS-SVM), and generates synthetic instances for improved representation of minority classes. The SCOTE algorithm splits multi-class datasets into binary imbalanced datasets, filtering noisy data points using a k-nearest neighbourhood (k-NN) method. Subsequently, samples are trained using LS-SVM, and minority samples are sorted based on their importance, determined by the misclassification error of minority classes in the training sets. Synthetic samples are then generated considering the decision region and the importance of data points, with a focus on enhancing diversity within the decision boundary.

In 2021, Gao et al. introduce a novel approach known as Differential Partition Sampling Ensemble (DPSE), which addresses the multiclass imbalanced dataset classification problem through the use of the One-Versus-All (OVA) decomposition strategy (57). This hybrid method combines re-sampling techniques with ensemble learning, aiming to enhance overall accuracy. By partitioning the original multi-class imbalanced dataset into individual binary datasets under the OVA framework, DPSE identifies instances as safe, borderline, or rare, using neighbourhood information. Differential sampling methods, such as s-Random under-sampling and br-SMOTE, are then applied to improve balance and diversity within each binary dataset. Subsequently, learning classifiers are trained on these balanced binary datasets, resulting in an ensemble of binary classifiers. The outputs of these classifiers are aggregated based on “Max strategy” (67) to determine the outcome.

A hybrid re-sampling algorithm, Similarity Over-sampling and Under-sampling Preprocessing (SOUP) is introduced in 2019 by Janicka et al. (54). This hybrid re-sampling method combines both under-sampling and over-sampling techniques within a unified framework. SOUP incorporates the concept of “safe level”, prioritising safe minority instances for synthetic generation during over-sampling while removing problematic majority class instances during under-sampling. The determination of safe levels is based on the degree of similarity, measured using the Heterogeneous Value Difference Metric (HVDM). Additionally, the algorithm establishes an order of execution to minimise the impact of changing safe levels, conducting under-sampling on majority instances from largest to least impact, and oversampling minority instances in the reverse order.

A unique paper that discusses binarisation strategies with classification algorithms is presented by Zak and Wozniak (55). The author discusses the usage of binarisation strategies such as “One-Vs-All” (OVA), “One-Vs-One” (OVO) and “Error-Correcting Output Codes” in addressing multi-class classification. The experimental findings demonstrate an enhanced performance of classifiers on multi-classification tasks, with the OVO binarisation strategy yielding the most robust results.

An analysis paper is published in 2016 by Saez et al. (52). This paper analyses the over-sampling of different classes and types of examples in multi-class imbalanced datasets. While not introducing new data pre-processing methods or classification algorithms, the paper aims to enhance understanding and improve outcomes of existing approaches. It proposes to select over-sampling techniques based on dataset structure analysis, defining instances as “safe”, “borderline”, “rare”, or “outlier”. This classification informs the selection of over-sampling strategies, tailored to the dataset's characteristics.

A LvH binarisation technique is proposed in comparison to traditional OVO and OVA techniques by Patil and Sonavane and published in 2017 (53). The LvH method compares individual lowest minority class against one highest majority class, reducing the replication of minority synthetic instances and improving computational efficiency. The authors incorporated LvH into newly proposed algorithms MEMMOT, MMMmOT, CMEOT and showed that it led to improved F-measure and ROC area score.

Pristyanto et al. propose to combine the usage of ADASYN (Adaptive Synthetic Oversampling) and stacking methodology to create a robust approach to improve classification performance in multi-class datasets (58). The authors incorporated the concept of meta-classifiers, which ingests the predictions of base classifiers (e.g., SVMs, Decision Tree) as inputs to derive at the final prediction.

In 2023, Liu et al. introduced Noise-robust oversampling techniques (NROMM) (59). It combines the clustering with adaptive sampling. Specifically, it employs the CFSFDP clustering techniques (68) to categorise each datapoint into inland sample, borderline sample, and trapped sample, thereby defining the class boundaries. After these boundaries that are unclear, NROMM removes both borderline, and trapped samples from majority classes. This enlarges between-class boundaries. Subsequently, synthetic samples are generated adaptively, within the safe boundary between classes. NROMM enhances the overall data structure and therefore improve classification accuracy.

In 2023, Ensemble-based Evidential Hybrid Re-sampling (E-EVRS) method is introduced (60). It utilises belief function theory to represent ambiguities such as overlapping and outliers. Bagging ensemble is performed to combine various decision boundaries, thereby encourage diversity. Subsequently, minority samples are generated towards the boundaries to strengthen the minority class boarders and reduce misclassification of difficult samples.

In the same year, OOSI (oriented oversampling with spatial information) is introduced (61). It adaptively partitions the data space by clustering methods based on database characteristics but disregard data labels. Within these clusters, synthetic data are generated according to the imbalanced ratio, distribution of minority samples, intra-cluster distance deviation, and multi-class density information. This method prevents excessive generation in unnecessary regions, ensures safe synthetic minority samples, and reduces the risk of introducing noise.

In 2023, Chui et al. (63) proposed ASMOTE-ENN, a hybrid methods that combines adaptive SMOTE (ASMOTE) with edited nearest neighbours (ENN) data cleaning technique. During the ASMOTE phase, synthetic minority instances are generated using SMOTE adaptively, adjusted with a random scaling factor. Subsequently, the ENN process cleans the data by evaluating each point against its K-nearest neighbour. If a data point and the majority of its nearest neighbours belong to different class, the point is removed. This step is repeated to ensure each class is appropriately represented and noise is reduced in the dataset.

In 2023, SMOTE-GAN-VAE is proposed (62), a three-stage data generation process for multi-class datasets. SMOTE generates synthetic samples, which are then refined by GAN theory to control data quality, minimise bias and maximise diversity. Following this, a VAE is employed to transform higher-dimensional data into lower-dimensional data. Specifically, the GAN step is crucial for high imbalanced dataset, as it encourages diversity, and hence improves the classification performance.

## 4 Discussions

### 4.1 Category summary

**Metric-based over-sampling** focuses on utilising innovative metrics for synthetic data generation. Distance metrics from other research fields have been applied to multi-class resampling, such as Hellinger Distance-based, HVDM distance measure, and EMDO. The Hellinger Distance-based is employed to direct the generation of synthetic samples towards areas with a higher concentration of neighbours within the same class (28). By leveraging HVDM as a distance measure, the algorithm dynamically adjusts its over-sampling strategy based on the differences between instances (29). The incorporation of MDO (30), AMDO (34) and EMDO (36) allows for multiple ellipsoids, which enables providing a more accurate estimation of the decision region of the minority class.

Additionally, new concepts from other research fields have applied to the data generation process for multi-class re-sampling. CCR algorithms leverage an energy-based approach for tidying up majority instances within spheres, which enables precise minority over-sampling (31). MC-RBO utilises potential disparities between classes to identify safe regions for over-sampling to generate synthetic instances (30). It employs a non-nearest neighbours' method, addressing unusual data distributions. It introduces the concept of “potential” based on a Gaussian RBF to identify regions of overlap or uncertainty between classes. In the CWGAN algorithm (32), the incorporation of Gradient Penalty as a regularisation tool, ensures a smooth data generation process within the decision region.

**Adaptive over-sampling techniques** dynamically adjust their strategies based on local data distributions, focusing on areas like boundary regions, and employing sophisticated measures like kd-trees and fuzzy neighbourhood-based feature selection.

LAMO dynamically adjusts the over-sampling process by identifying borderline minority instances using local Euclidean distance distributions (37). It adapts the over-sampling strategy based on the significance of each candidate minority instance, ensuring the generation of synthetic instances aligns with their distribution. SMMO progressively generates new minority instances (40), chosen based on specific criteria. These newly generated instances are then utilised in the generation of subsequent instances. AFNFS introduces an adaptive fuzzy neighbourhood radius based on margins of similar and dissimilar instances, adjusting the over-sampling model to achieve a balanced environment of synthetic and original instances (38). The algorithm dynamically determines the fuzzy neighbourhood radius and fuzzy neighbourhood granule based on specific dataset characteristics, making the over-sampling strategy flexible and adaptive. OREM incorporates boosting techniques to dynamically adjust the over-sampling of minority class instances during each iteration of the boosting process. By focusing on the detection of clean sub-regions within candidate minority regions, the algorithm adaptively generates synthetic instances to expand the minority region reliably (39).

**Structure-based over-sampling** techniques focus on leveraging the underlying structure and distribution of data. They normally incorporate methods such as KNN, clustering and data distribution analysis to determine the suitable regions for generating synthetic instances. The techniques aim to address issues such as over-generalisation, boundary behaviour, and data hierarchy to guide the over-sampling process.

Many research papers falls under this category utilise clustering or nearest neighbours concept to identify data structure, such as SMOM (22), SOMCID (42), PAIO (48). UCPMOT (41), KNSMOTE (45), PBO (47), MuDIOT (43), CS (49). In addition, Deng et al. presented a method that brings an integration of data sorting based on distance to the hyperplane, composite weight-driven sampling decisions, intra and inter-class sampling strategy at class boundaries, and utilisation of neighbourhood information to capture data density (46). Li et al. incorporates spectral clustering to partition minority classes into subspaces (44). Gaussian Mixture Model is employed to estimate the class distribution (50). SMOTE-IF incorporates Isolation Forest technique to identify and remove erroneous samples (51).

**Hybrid over-sampling methods** blend elements from diverse techniques to effectively tackle imbalanced data challenges. One of the most prominent groups of hybrid re-sampling methods aims to integrate concepts from multiple over-sampling techniques or approaches. Techniques include: SCOTE combined with LS-SVM for imbalanced bearing fault diagnosis (56); NROMM combines the clustering with adaptive sampling (59); E-EVRS (60) combines belief function theory to represent overlapping and outliers with bagging ensemble to combine various decision boundaries; OOSI (61), combines adaptive learning with clustering methods; ASMOTE-ENN (63) combines adaptive data generation with data cleaning techniques. SMOTE-GAN-VAE (62) combines data sampling techniques with data cleaning, and dimension reduction data to encourage diversity.

Another group of hybrid re-sampling methods focuses on incorporating ensemble methods with differential sampling to improve classification accuracy across multiclass datasets: DPSE method utilises differential sampling methods such as s-Random under-sampling and br-SMOTE over-sampling within the one-vs.-all (OVA) framework (57). SOUP algorithm combines over-sampling and under-sampling techniques based on the similarity of class distributions (54).

Furthermore, some hybrid methods aim to introduce a more comprehensive view of re-sampling techniques, such as binarization strategy, and data structure analysis. Zak and Wozniak combine binarisation strategies with classification algorithms to assess their impact on classifier quality in addressing multi-class classification challenges (55). Patil and Sonavane also integrated binarization techniques with traditional over-sampling methods (53). Saez et al. focuses on understanding over-sampling approaches without introducing new preprocessing methods, emphasising dataset structure analysis for effective classification improvement (52).

### 4.2 Citation analysis

Metric-based over-sampling category has garnered a total of 905 citations, with AMDO (34) receiving the most citations (378) within this group.

Adaptive over-sampling category has accumulated 108 citations. The earliest paper published in this category LAMO in 2021 (37) receives the most citations (65).

In structure-based over-sampling category, which has a total of 522 citations, of these, the earliest publication in 2017, SMOM (22), has the most citations (234).

Hybrid method category has a total of 516 citations. The earliest publication in 2016 (53) has the most citations (250).

### 4.3 Trend analysis

**Metric-based over-sampling:** there is a trend where increased attention is devoted to understanding similarity or differences during the resampling process as it can have significant influence. During the data preprocessing stage, methods focus on refining decision regions through the incorporation of diverse distance measures. This refinement aids in noise reduction and facilitates the identification of overlapping regions, which is crucial in multiclass scenarios. Another notable advancement involves the utilisation of alternative metrics during the resampling process, such as employing information gain, which is a novelty in the context of oversampling techniques.

**Adaptive over-sampling:** the rise of advanced and highly efficient computing power has enabled the emergence of adaptive techniques, which are more sophisticated and reactive relative to the traditional oversampling approaches. Unlike conventional approaches, adaptive techniques can operate iteratively, and dynamically adjust the overall structure of the dataset. The ability to confront hard-to-determine instances accordingly with these iterative and dynamic approaches, allows targeted intervention, which generates more representative synthetic instances. This thereby assists in enhancing the predictive capabilities of subsequent classification or learning models.

**Structure-based over sampling:** due to the intricate nature of multi-class datasets, careful handling is essential during the process of resampling. A detailed, extensive understanding of the structure of the underlying dataset, knowledge of complex inter-relationship and intra-relationship of classes is paramount. This can be achieved using techniques such as clustering or KNN to locate the safe region for each minority class instance. Valuable insights can also be derived by factoring in the relative data positioning and identifying the appropriate safe threshold. Moreover, the learning of dataset structure serves to expand decision regions and prevent wrongful treatment of minority class instances into outliers.

**Hybrid over-sampling:** these methods have gathered popularity in the literature for their efficacy in combining advantages from diverse processes. Specifically, majority of 2023 papers fall under this category. These methods cover a range of approaches, including combining different over-sampling techniques, integrating over-sampling with under-sampling, merging resampling with enhanced classifiers, or employing varied binarisation. The ability to leverage the strengths of multiple methods enables hybrid approaches to be a flexible toolkit for tackling the intricacies of imbalanced data. Recently, there has been a notable rise in the practice of performing data cleaning after data generation, motivated by the need to establish clearer boundaries, a particular challenge in multi-class scenarios.

## 5 Conclusions and future research

In medical classification tasks, multi-class classification provides more detailed information compared to binary classification, but it also introduces more complexity, particularly in re-sampling. This is due to issues of smaller sample sizes and more pronounced class overlaps, driven by complex interactions between classes. This situation requires sophisticated synthetic data generation to represent minority classes without overlapping. To tackle such a challenge, advanced algorithms for equitable instance creation among diverse classes are needed. While there has been notable progress, multi-class imbalance re-sampling is still in its infancy, presenting many opportunities for further research in this field.

The exploration of over-sampling strategies across metric-based, adaptive, structure-based, and hybrid methods outlines the comprehensive effort in addressing the challenges of imbalanced datasets in the context of machine learning. Each category of approaches brings forth the intention to enhance data representation and improve classification accuracy. Metric-based methods focus on distance, or other innovative measures to guide synthetic sample generation. Adaptive approaches dynamically adjust over-sampling based on data distribution characteristics. Structure-based techniques depend on gathering knowledge of the inherent data structure for sample generation. Hybrid methods combine multiple strategies to offer versatile solutions. Together, these approaches signify a maturing field, which provides a solid bedrock for future innovations which will continue to deliver more effective and promising solutions for imbalanced data challenges.

For future research, in health informatics, it is recommended to enhance the following:

1. Metrics: Identifying and utilising novel metrics and techniques from binary classification, and more generally, the novel metrics introduced in Artificial Intelligence. Hellinger distance, Evolutionary Mahalanobis Distance have been brought into over-sampling strategies. There could be other distance metrics that can be employed, and their performance should be evaluated. For instance, Canberra distance is suitable for measuring distance for sparse data. By incorporating and applying new distance measures, researchers can potentially improve the accuracy and effectiveness of over-sampling methods in addressing class imbalance.
2. Adaptive Methodology: Since 2020, there has been a noticeable trend towards integrating adaptive methodologies into resampling techniques. This is particularly crucial within the field of medical classification as the dataset often encounters data quality issues due to frequent manual collation of data and information. By iteratively addressing the most challenging instances, this approach enhances classification performance. In guiding future research efforts, numerous advancements can be explored in this field, ranging from pre-resampling techniques for iterative outlier cleanup to in-process adaptations for dynamically generating data points based on updated data structures.
3. Binarisation Strategy: As the literature transitions from one-vs.-one (OVO) to one-vs.-all (OVA) frameworks, it becomes evident that OVA is better suited for multi-class classification tasks, given its focus on the entire data structure encompassing all classes rather than just two. To guide future research, OVA can be effectively integrated into various contemporary binary resampling methods. Further exploration is recommended to identify and evaluate binary resampling methods that are particularly well-suited for multi-class scenarios under the OVA binarisation framework.
4. Hybrid Technique Development: For future research, it is recommended to explore combinations of diverse techniques, encompassing various metrics, methodologies, and machine learning techniques, such as cost-sensitive learning and ensemble methods. By integrating these elements, researchers can develop more resilient oversampling solutions aimed at improving overall prediction accuracy and effectiveness, particularly in multi-class imbalanced scenarios.
5. Increasingly, the importance of data structure has gained huge prominence. In the field of health informatics, extremely imbalanced datasets are common and therefore there is very limited representation of minority instances. This implies that utilisation of diversity-based metric within the specified extremely imbalanced minority classes, while retaining the underlying data structure is important in ensuring that there is sufficient variability and variation in the synthesised dataset.
6. Scalability and Efficiency Enhancement: This is not trivial as medical datasets typically consists of many variables and are often presented in large sizes. Therefore, efficiency is particularly important in the process of re-sampling. Given the efficiency challenges posed by the complexity of algorithms and the presence of multiple classes, this research direction can focus on optimising over-sampling approaches for better performance in large-scale datasets. The goal is to ensure that these techniques remain practical and effective even as data volume and complexity grow.
